# Dynamic architectural interplay between leucocytes and mammary epithelial cells

**DOI:** 10.1111/febs.15126

**Published:** 2019-11-29

**Authors:** Jessica R. Hitchcock, Katherine Hughes, Olivia B. Harris, Christine J. Watson

**Affiliations:** ^1^ Department of Pathology University of Cambridge UK; ^2^ Department of Veterinary Medicine Cambridge UK

**Keywords:** imaging, involution, leucocytes, mammary gland

## Abstract

The adult mammary gland undergoes dynamic changes during puberty and the postnatal developmental cycle. The mammary epithelium is composed of a bilayer of outer basal, or myoepithelial, cells and inner luminal cells, the latter lineage giving rise to the milk‐producing alveolar cells during pregnancy. These luminal alveolar cells undergo Stat3‐mediated programmed cell death following the cessation of lactation. It is established that immune cells in the microenvironment of the gland have a role to play both in the ductal outgrowth during puberty and in the removal of dead cells and remodelling of the stroma during the process of postlactational regression. However, most studies have focussed on the role of the stromal immune cell compartment or have quantified immune cell populations in tissue extracts. Our recent development of protocols for deep imaging of the mammary gland in three dimensions (3D) has enabled the architectural relationship between immune cells and the epithelium to be examined in detail, and we have discovered a surprisingly dynamic relationship between the basal epithelium and leucocytes. Furthermore, we have observed morphological changes in the myoepithelial cells, as involution progresses, which were not revealed by previous work in 2D tissue sections and whole tissue. This dynamic architecture suggests a role for myoepithelial cells in the orderly progression of involution. We conclude that deep imaging of mammary gland and other tissues is essential for analysing complex interactions between cellular compartments.

## Graphical abstract

In this study by Christine Watson and colleagues, deep 3D‐imaging was used to investigate changes in the architecture of myoepithelial cells and leukocytes in mammary ducts and lobuloalveolar structures. Leukocytes intercalate between ductal myoepithelial and luminal cells and surprisingly, closely associate with myoepithelial cells during lactation and involution, adopting their shape. Myoepithelial cells contract progressively as involution proceeds and shrink back towards the ducts, possibly providing a barrier to the dissemination of milk and dying luminal cells.

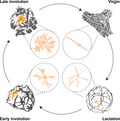

**Abbreviations**
2D: two dimensions
3D: three dimensions
CD: cluster of differentiation
DAPI: 4′,6‐diamidino‐2‐phenylindole
DCIS: ductal carcinoma *in situ*
DCs: dendritic cells
DNA: deoxyribonucleic acid
GPI: glycosylphosphatidylinositol
H&E: haematoxylin and eosin
IgA: immunoglobulin A
K5: keratin 5
LIF: leukaemia inhibitory factor
MHCII: major histocompatibility complex class II
MIP: maximum intensity projection
MMPs: matrix metalloproteinases
NF‐κB: nuclear factor kappa‐light‐chain‐enhancer of activated B cells
SEMA: semaphorin
SMA: smooth muscle actin isoform α‐actin
Stat: signal transducer and activator of transcription
TEBs: terminal end buds
Th: T helper
VEGF: vascular endothelial growth factor

## Introduction

The mammary gland is somewhat unusual in that most of its development takes place in the adult, with newborn mammals having a simple rudimentary branched network of ducts emanating from the nipple region 1. Subsequently, the gland undergoes distinct phases of development primarily in response to hormones. At the onset of puberty, large club‐shaped structures called terminal end buds (TEBs) arise at the tips of the ducts and invade the fat pad, elongating and bifurcating until the limits of the fat pad are reached whereupon they regress to be replaced by terminal duct structures. In adult mice, the mammary gland undergoes a cycle of proliferation and regression with each ovarian cycle in response to changing levels of oestrogen and progesterone 2, 3. During dioestrus, following ovulation, alveolar buds form on secondary branches and are subsequently removed if pregnancy does not occur 2. Pregnancy hormones induce another phase of rapid growth marked by secondary branching and the formation of lobuloalveolar structures at their tips which differentiate to produce milk during lactation. Upon the cessation of lactation, the alveolar structures are removed by programmed cell death 4, 5, 6, 7, 8 with concomitant tissue remodelling. Stromal reorganisation involves enzymes such as matrix metalloproteinases (MMPs) 9, with MMP2, MMP3 and MMP9 downregulated in the absence of epithelial Stat3 signalling 10.

The mammary epithelium comprises a simple branched bilayer of basal myoepithelial cells that contact the stroma and support an inner layer of luminal cells that line the duct 1. These cells can be distinguished microscopically by their shape and by expression of specific intermediate filaments that allow them to be unambiguously identified. For example in mice, keratin 5 (K5) and keratin 14 (K14) are expressed in only the basal lineage, while keratin 8 (K8) and keratin 18 (K18) are restricted to the luminal lineage and are thus utilised as surrogate markers of their respective lineages 11. The luminal cells may also be delineated by their expression of E‐cadherin, while the smooth muscle actin isoform α‐actin (SMA) can be used to identify basal cells 12. In two‐dimensional studies in other rodent species, the myoepithelium can also be seen to exhibit morphological changes with the mammary postnatal developmental cycle, including extension of processes during pregnancy and cytoplasmic vacuolation after weaning 13, 14. A fully involuted gland morphologically resembles a virgin gland although it has a higher density of side branches 15.

Throughout these dramatic cycles of growth and tissue reorganisation, the stroma and the immune cell compartment are dynamically altered and immune cells vary in frequency and type at different mammary postnatal developmental time points. Macrophages have been demonstrated to play a critical role in the mammary microenvironment during postnatal development, including regulation of branching morphogenesis. While macrophages, eosinophils and mast cells surround the TEBs that arise during puberty, T and B lymphocytes are notably absent 16, 17, 18. In nonpregnant female mice, the highest numbers of macrophages are present in dioestrus although a larger number are in direct contact with the epithelium during pro‐oestrus 19, 20, 21. Macrophages also play a fundamental role during mammary postlactational regression 10, 21, 22, 23 when active Stat3 signalling in the mammary epithelium is critical in the development of an immunomodulatory phenotype in these cells 24. This contributes to the overall acquisition of a ‘wound healing’ signature in the mammary gland 25, 26. Interestingly, recent studies have demonstrated that fetal‐derived macrophages predominate in mammary gland stroma of the adult 27 while NOTCH‐expressing macrophages have been shown to interact with basal mammary stem cells expressing the delta‐like 1 NOTCH ligand to promote their expansion 28. Thus, proper postnatal development of the mammary gland requires an associated population of tissue‐resident macrophages.

Similar to macrophages, eosinophils fulfil an important role in mammary gland development, both in mice 16 and in species such as cows that possess terminal ductulolobular units rather than TEBs 29. Eosinophil recruitment to the prepubertal mouse mammary gland coincides with a dramatic increase in eotaxin mRNA transcript levels, and, like macrophages, eosinophils are thought to promote branching and formation of TEBs 16, 17. Mast cells also exhibit fluctuations in number during different stages of mammary postnatal development 30, 31, and numbers present during murine involution are influenced by mammary epithelial Stat3 signalling 10, 23. Elaboration of plasma kallikrein, a plasminogen activator, may be one role of the connective tissue‐type mast cells associated with postlactational regression 18. The mammary immune microenvironment also includes CD3‐positive T lymphocytes 32, and the presence of plasma cells has been demonstrated 25, correlating with historical studies highlighting the presence of IgA in the mammary gland during involution 33. Recently, the similarity between the mammary gland and mucosal tissues has been emphasised and the presence of populations of functionally distinct subtypes of T lymphocytes and dendritic cells has been delineated 34.

To date, the majority of studies of immune cells in the mammary gland have relied on flow cytometry and histological studies of tissue sections, and while these have provided insights into the numbers and identity of immune cells present in mammary tissue, they have not provided detailed information on their relationship with the luminal or basal epithelium nor their architectural arrangement within the tissue. We sought to address these questions by utilising our recently developed tissue clearing and deep tissue imaging protocols based on those developed by Susaki *et  al*. 35, 36, 37, 38, 39. Our observations revealed a surprisingly dynamic relationship between immune cells and the myoepithelial compartment and the intimate connections between the vascular and ductal epithelial networks.

## Results

Our recent optimisation of tissue clearing and imaging of the mammary gland enables *in situ* visualisation of the ductal system and its surrounding stroma in three dimensions (3D) 36. We sought to use these approaches to investigate epithelial morphogenesis throughout a pregnancy/lactation/involution cycle. Here, we consider this tissue remodelling in the context of the intact mammary stroma, focussing on immune cells and their interplay with the epithelial network.

### Association of CD45+ cells with the mammary epithelium in virgin mice

**Figure 1:**
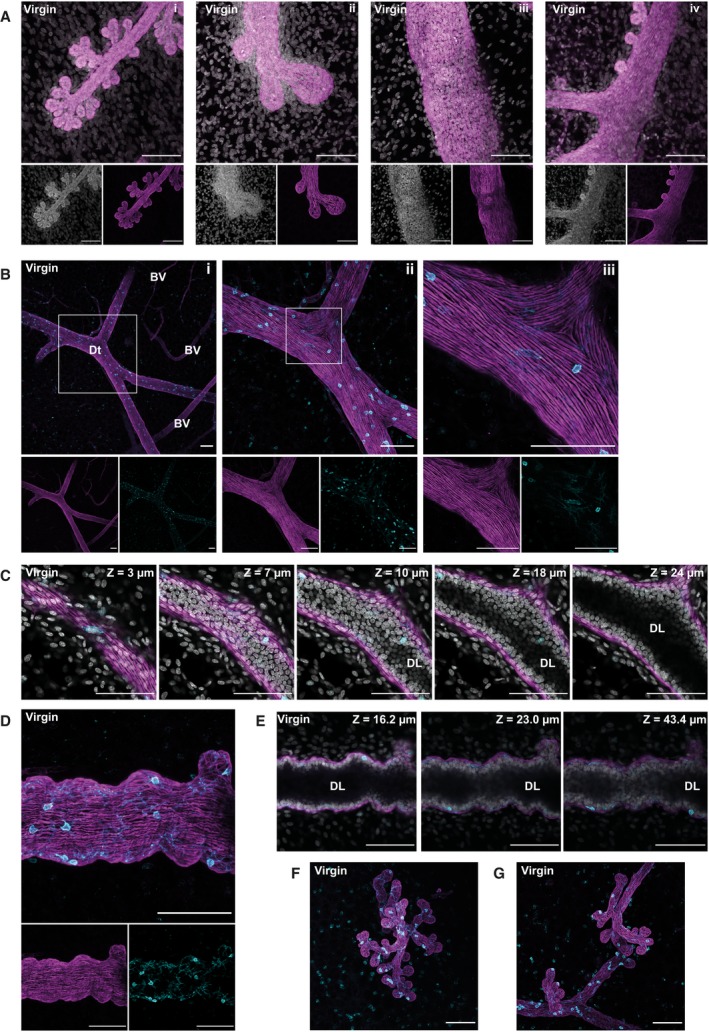
Leucocytes localise to mammary ducts and reside in the intraepithelial bilayer. Three‐dimensional (3D) confocal microscopy of optically cleared virgin mammary glands from BALB/c mice immunostained for the myoepithelial cell marker smooth muscle α‐actin (SMA) (magenta) and the pan‐leucocyte marker CD45 (cyan), and nuclei were stained with DAPI (grey). (A) Three‐dimensional maximum intensity projections (MIPs) of the entire image sequence captured where the larger panels (i–iv) show the merge of individual SMA and DAPI stains (smaller panels); (B) MIPs of a primary mammary duct, with single stains shown below the main panel. Higher magnification images of the boxed region are shown in each subsequent panel (i–iii); (C) 5 individual optical slices (0.68 μm thick), through a stack with the depth (*z* value) relative to the first image in the sequence; (D) MIPs of a duct; individual stains shown in the panels below; (E) individual optical slices (0.68 μm thick) through the optical stack shown in (D); the depth (*z* value) is relative to the start of the image sequence; (F, G) MIPs of the entire image sequence captured. Images are representative of seven mice; all scale bars represent 100 μm. Dt, mammary duct; BV, blood vessel; DL, duct lumen.

We initially examined whole mammary tissue from adult virgin mice, in which the ductal system is fully expanded to fill the fat pad and TEBs have regressed. Maximum intensity projection (MIP) of SMA‐stained glands highlighted the varying extent of ductal side branching and alveolar budding that is observed in postpubertal mice (Fig. 1A). Although 2D imaging can be sufficient to demonstrate broad changes in branching morphogenesis and alveolar budding across the oestrous cycle, as has been shown previously 40, here we highlight the importance of deep imaging analyses that do not depend critically on the plane of section and where the relationship between buds and branches is much more visually apparent. We noted also the precise orientation and high density of the long, thin basal myoepithelial cells that run in parallel to the direction of ductal elongation (Fig. 1B iii). This organised arrangement may provide strength and elasticity to the ducts enabling their expansion when they are engorged with milk during lactation. Notably, the myoepithelial cells are reorientated at branch points and at the tips of branches (Fig. 1A,B).

Immune cells have previously been described in the stroma, closely associated with the ductal epithelium and particularly at the tips of growing ducts, around the TEBs. However, numbers of many of these cells, including eosinophils and mast cells, decline in parallel with the regression of the TEBs 41. Furthermore, fluctuations in oestradiol and progesterone during an ovarian cycle have been shown to influence immune cell populations in the mammary gland 20. Thus, we sought to determine the distribution of immune cells in fully developed adult mammary glands of virgin mice using the pan‐leucocyte marker CD45 (lymphocyte common antigen). We noted a striking distribution of CD45+ cells that appear to be distributed throughout the ductal epithelium (Fig. 1B) with individual cells exhibiting an intimate relationship with the basal epithelium (Fig. 1B ii,iii). On closer inspection using sequential optical slices, it became apparent that CD45+ cells are intercalated between the epithelial bilayers of the ducts (Fig. 1C–E). Importantly, CD45+ cells are rarely observed within the lumen of the ducts. Of note, many of the CD45+ cells observed in virgin mice have a dendritic morphology (Fig. 1D) with processes frequently stretching along the interface between the basal and luminal layers (Fig. 1E).

DAPI staining of nuclear DNA highlights the large number of cells (both CD45 positive and negative) within the stroma, including adipocytes, fibroblasts, and cells comprising the blood and lymphatic vessels (Fig. 1A,C). Although SMA also delineates the vascular network (Fig. 1Bi), the distinctive arrangement of the actin network, which encircles these vessels rather than running along their length, allows them to be clearly distinguished from the mammary ducts.

We also noted that each individual side branch or bud frequently has at least one associated CD45+ cell (Fig. 1F,G). Previous work showed that CD11c+ cells (encompassing dendritic cells and a subpopulation of macrophages) play an inhibitory role in branching morphogenesis 42. This suggested to us that CD45+ cells could be important components of the ductal architecture during a pregnancy cycle, and this prompted us to investigate the distribution of immune cells in lobuloalveolar structures during lactation.

### Association of CD45+ cells with the mammary epithelium in lactating glands

**Figure 2:**
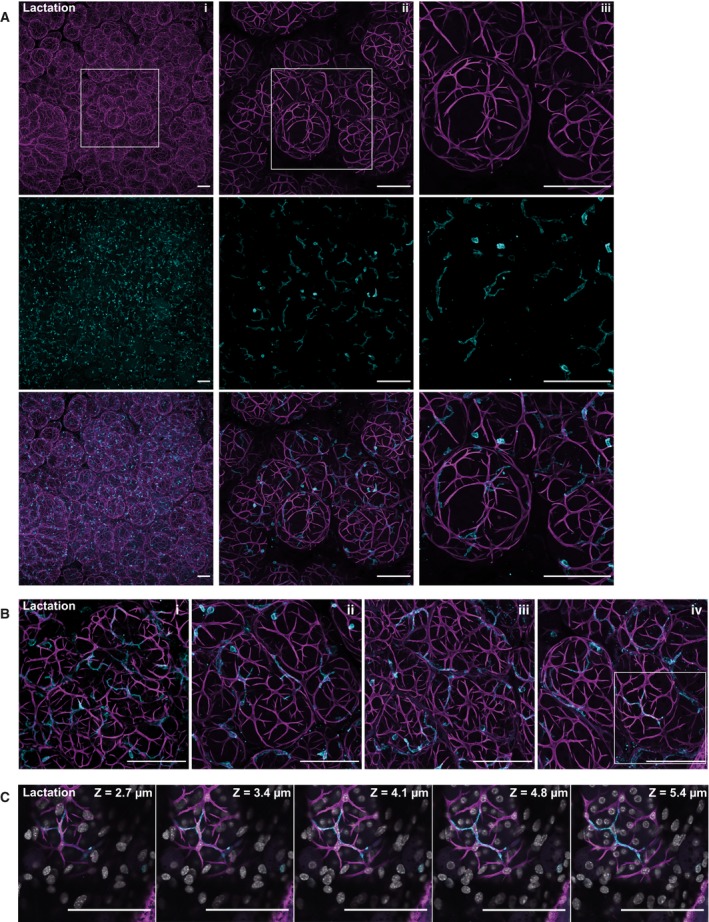
The distinct basal epithelial cell morphology in lactation is shared by the associated leucocytes. Mammary glands from lactating BALB/c mice were optically cleared and immunostained for smooth muscle α‐actin (SMA) (magenta) and CD45 (cyan), and nuclei were stained with DAPI (grey). (A) Three‐dimensional maximum intensity projections (MIPs) of the entire image sequence showing myoepithelial cells (top row), leucocytes (middle row) and a merged image (bottom row). Higher magnification images of the boxed region are shown in each subsequent panel (i–iii); (B) MIPs of the entire image sequences of four individual alveolar clusters, each from a different mouse, showing overlap between the morphology of the SMA and CD45‐positive cells; (C) 5 sequential optical slices (0.68 μm thick) of the area shown in the boxed region in B (iv), where the depth (*z* value) is relative to the first image in the sequence. Images are representative of a total of six mice; all scale bars represent 100 μm.

Pregnancy results in a massive expansion of the mammary epithelium, and the fat pad becomes filled with lobuloalveolar structures that cluster at the tips of tertiary branches. At the onset of lactation, a final round of proliferation takes place and the majority of mammary epithelial cells then exit the cell cycle and become secretory milk‐producing cells 1. Immunostaining for SMA revealed that the basal cells surrounding alveoli adopt a completely different shape to those in the ducts with a central nucleus and cytoplasmic extensions forming a stellate shape (Fig. 2A i,ii and iii) 36. Several myoepithelial cells encompass each lobule, forming a basket‐like network 35. This arrangement of myoepithelial cells is necessary for their contraction (in response to oxytocin) to expel milk into the ducts 43, 44. Indeed, genetic deletion of the SMA‐alpha 2 isoform encoded by the *Acta2* gene results in diminished contractility of myoepithelial cells and reduced ejection of milk 45.

Immunostaining for CD45+ cells revealed that these cells have a different morphology to those in the ducts of virgin mice. During lactation, many leucocytes seem to be closely associated with the myoepithelial cells; the cells appear to colocalise, often lying adjacent to one another and adopting the same shape (Fig. 2A,B). Sequential thin optical sections through a single alveolus (Fig. 2C) show that CD45+ cells appear to intercalate between the overlying basal cells and the luminal cells, which are frequently binucleated in the lactating mammary gland 32, 46, 47. A comparison of cells in the virgin ducts with those in the alveoli (compare Fig. 1B ii with Fig. 2A ii) reveals a striking increase in the preponderance of CD45+ cells with a dendritic‐like morphology during lactation, the majority of cells exhibiting extensive, fine cytoplasmic processes. In other tissues, it is well established that dendritic cells can extend their cytoplasmic processes through even the tight junctions between epithelial cells to sample antigen. Thus their localisation between the myoepithelial and luminal layers, and the increased frequency of dendritic‐shaped cells during lactation, may reflect enhanced immunosurveillance at this time.

### Dynamic association and rearrangement of CD45+ cells during involution of the mammary epithelium

At the cessation of lactation, a programme of cell death, coupled with tissue remodelling and redifferentiation of the fat pad adipocytes, is initiated 48. This process ultimately returns the gland to a near prepregnant state. Within 12 h of forced involution, luminal epithelial cells are stochastically induced to undergo programmed cell death, and by 72 h after forced involution, the alveoli are visibly collapsing and lipid‐filled adipocytes are apparent 49. It is well established that involution is associated with an influx of macrophages and other immune cell types that are thought to be an essential component of the involution process 22. However, involution has not been visualised in 3D, and thus, we therefore carried out an analysis of involution at five time points over an 11‐day time course with a focus on the basal epithelial and CD45+ cells, their morphology and their localisation.

**Figure 3:**
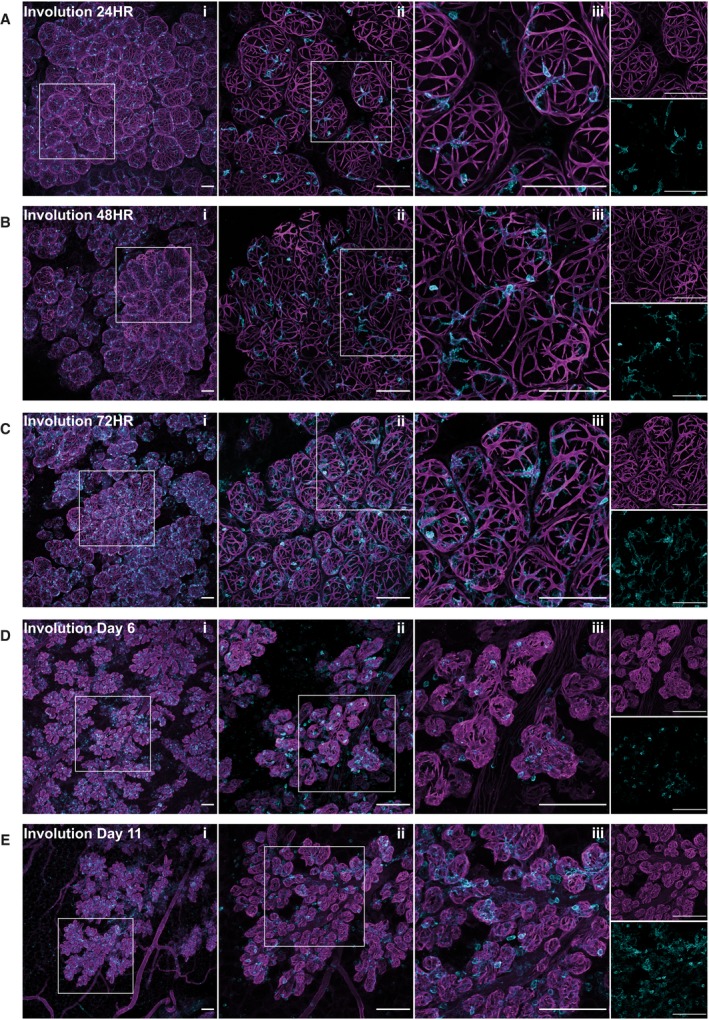
Leucocytes accumulate in the mammary gland during involution. Mammary glands from force‐involuted BALB/c mice were harvested, optically cleared and immunostained for smooth muscle α‐actin (SMA) (magenta), CD45 (cyan) and DAPI (grey). Three‐dimensional maximum intensity projections (MIPs) of the entire image stack captured are shown for mice harvested at (A) 24 h, (B) 48 h, (C) 72 h, (D) 6 days and (E) 11 days after forced involution. Panels i, ii and iii show merged SMA‐ and CD45‐stained images; boxed regions in panel i are enlarged in panel ii; boxed regions in panel ii are enlarged in panel iii; individual stains of the merged images in panel iii only are shown. Images are representative of four mice (24 h), two mice (48 h), nine mice (72 h), four mice (6 days) and four mice (11 days) involution; all scale bars represent 100 μm.

At 24‐h involution, the architecture of the gland is very similar to that in lactation and lobules are fully expanded (Fig. 3A). The lumens of the alveoli at this time are characterised by accumulation of milk, milk fat globules and dying cells that have been shed from the alveolar structure. As involution progresses, subtle changes in the myoepithelial cells are observed at 48 h (Fig. 3B), the time when the progression to irreversible involution occurs 50. This slight condensing of the myoepithelial cells at 48 h is more apparent by 72‐h involution, where the collapse of the alveolar structures is obvious and the myoepithelial cells, while maintaining their shape, are shorter and more condensed (Fig. 3C). More dramatic changes take place subsequently, and by 6 days of involution, the actin filaments are highly condensed and disorganised and the alveolar structures are contracted, shrinking towards the ducts (Fig. 3D). By 11 days of involution, these condensed alveoli are still present, although they are substantially reduced in size (Fig. 3E).

The arrangement and morphology of CD45+ cells also changes as involution progresses. Notably, at 24 h, these cells are still associated with the myoepithelial cells and their morphology is very similar to that present during lactation (Fig. 3A). By 48‐h involution, while many leucocytes are dendritic in shape, their processes are shorter than those seen at 24 h, possibly reflecting the condensing of the basal cells of the contracting alveoli (Fig. 3B). At 72‐h involution, there are visibly more CD45+ cells and these associate with the alveoli, often, but not always, colocalising with myoepithelial cells (Fig. 3C). By 6 and 11 days of involution, the CD45+ cells are more round with fewer cytoplasmic processes and their morphology more closely resembles that of the CD45+ cells observed in the virgin gland (Fig. 3D,E). In order to examine these populations of cells further, we immunostained with various markers for specific types of immune cells.

**Figure 4:**
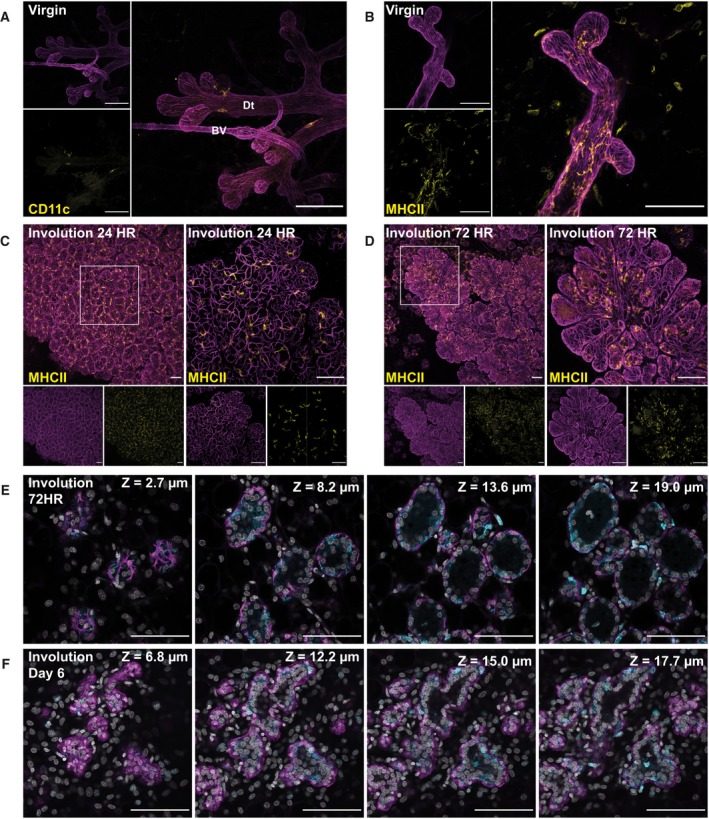
Leucocytes closely associate with the mammary epithelium during involution, residing between the luminal and basal alveolar cells. Mammary glands from BALB/c mice were harvested at the indicated time points after forced involution, optically cleared and imaged using confocal microscopy. Images in (A–D) show 3D maximum intensity projections (MIPs) of the entire sequence captured and were immunostained for smooth muscle α‐actin (SMA) in conjunction with (A) CD11c (yellow) and (B–D) MHCII (yellow); smaller images show individual stains of the merged image in the larger panels. (E, F) Individual optical slices (2.0 μm thick), from an optical stack (E = 72 h involution; F = 6 days of involution), where the depth (*z* value) is relative to the first image in each sequence. Glands were immunostained for SMA (magenta) and CD45 (cyan). Images are representative of four mice at each time point examined; all scale bars represent 100 μm; Dt, mammary duct; BV, blood vessel.

Considering the dendritic morphology of the CD45+ cells, we first looked for dendritic cells (DCs) using CD11c. However, we had observed in virgin mice that while these cells were present (Fig. 4A), they could not account for the majority of CD45+ cells observed. Thus, we hypothesised that most of the dendritic‐shaped CD45+ cells present during involution are likely to be macrophages. Unfortunately, none of the macrophage markers examined were compatible with the tissue clearing protocol, except for major histocompatibility complex class II (MHCII), which is upregulated on both activated DCs and macrophages. In virgin mice, the pattern of MHCII expression was similar to that seen using CD45 (Fig. 4B); therefore, we used MHCII expression for analyses during involution time points. At both 24‐ and 72‐h involution, MHCII+ cells are abundant within the alveolar structures and staining for MHCII closely resembles that seen for CD45 (Fig. 4C,D) although it does not fully account for all the CD45+ cells present.

To determine whether immune cells intercalate between the epithelial layers during involution, we examined individual z slices. At 72 h postweaning, CD45+ cells are predominantly found adjacent to the basal cells, suggesting they reside between the two layers (Fig. 4E). Notably, only very few CD45+ cells were observed within the alveolar lumen at this time. At 6 days of involution, immune cells are clearly observed in the alveolar lumen, in addition to their localisation in the intraepithelial space (Fig. 4F). This may indicate a loss of the integrity of the luminal layer or migration of the CD45+ cells into the luminal space where they could carry out a phagocytic function.

### Imaging in 3D reveals the multifocal nature of the involution process in mammary gland

**Figure 5:**
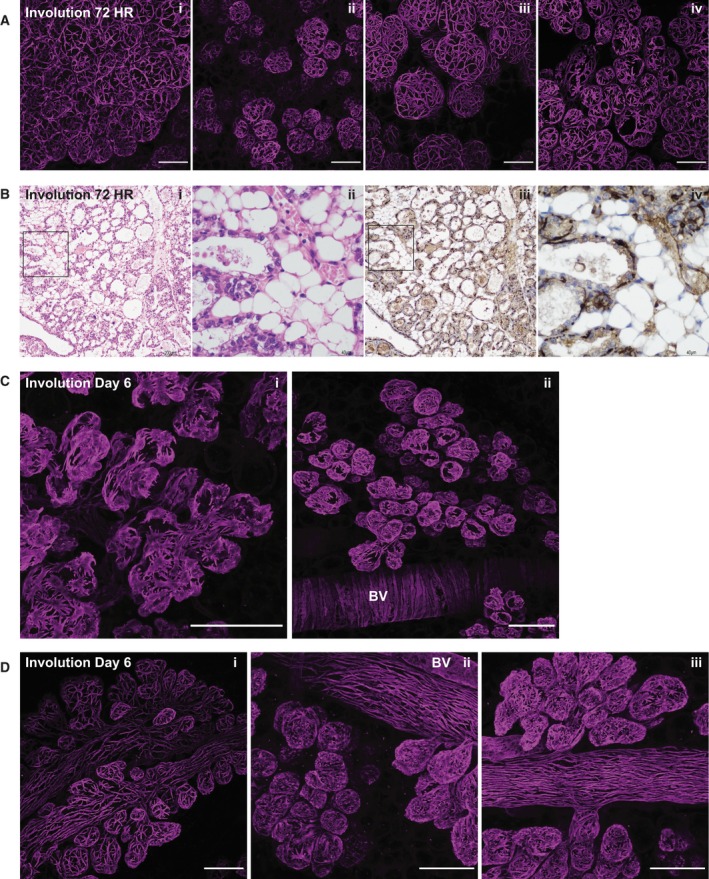
During involution, myoepithelial cells contract towards the ductal tree while maintaining the structure of the alveoli. Mammary glands from BALB/c mice were harvested at 3 and 6 days of involution, optically cleared and immunostained for the myoepithelial cell marker smooth muscle α‐actin (SMA) (magenta) and DAPI (grey). Mammary glands harvested from C57BL/6 mice 72 h after involution were processed by conventional histological techniques. (A) Three‐dimensional maximum intensity projections (MIPs) of the entire image stack of four individual alveolar clusters from four different mice (i–iv). (B) Tissue sections of mammary tissue at 72‐h involution were stained using H&E (i, ii) and anti‐SMA antibody (brown) with haematoxylin counterstain (iii, iv). Boxed areas are enlarged in the image to the immediate right. (C, D) MIPs of SMA (magenta)‐stained mammary glands at involution day 6 demonstrating the contraction of the alveolar clusters (C) and their close localisation to the ductal tree (D). Images are representative of nine mice (A) and four mice (B–D). Scale bars in A, C and D represent 100 μm. Scale bars in B represent 200 μm (i and iii) and 40 μm (ii and iv). BV, blood vessel.

The multifocal nature of involution is apparent even in a forced involution where suckling pups from normalised litters are removed at the peak of lactation. Figure 5A demonstrates the varying extent of involution progression that can be observed at 72 h within a single gland. Note the fully expanded lobuloalveolar clusters in (i) relative to the much more collapsed structures in (ii) and (iv). This might suggest that dying cells secrete a cytokine or other signal that acts in a paracrine manner to induce the death of other cells within the alveolar cluster. The specific activation of Stat3 within 12 h of forced involution, and the requirement of LIF 51 and subsequently oncostatin M 52 for this activation, would support this contention.

Myoepithelial cells are very difficult to see in H&E sections during lactation and involution (Fig. 5B i,ii) unless they are immunostained (Fig. 5B iii,iv), and it is practically impossible to detect the architectural changes in the myoepithelial cells during involution with such 2D imaging tools. Using our 3D imaging approach, myoepithelial cells are very easy to visualise and, for the first time, we have an understanding of their dynamic nature *in situ*. This facilitated the unexpected discovery that myoepithelial cells do not, in fact, die concomitantly with the luminal cells during involution. Instead, they change shape as the alveoli contract with loss of the luminal cells, in a previously unrecognised retreat towards the ductal tree (Fig. 5C) where elongated myoepithelial cells appear to become incorporated into the parallel strands of the ducts (Fig. 5D) or remain as small outpouchings (Fig 5D ii,iii).

This striking change in the size and shape of basal alveolar cells indicates that they may not be ‘neutral’ bystanders in the involution process. Presumably the mechanism of luminal cell death (whereby the uptake of milk fat droplets leads to lysosomal membrane permeabilisation) 5 does not occur in myoepithelial cells, and instead, these cells may realign with the ducts in preparation for a subsequent pregnancy. Since myoepithelial cell morphology provides a useful marker for the progression of involution, we suggest that involution studies could include 3D analyses to avoid any misinterpretation resulting from the multifocal nature of this remodelling programme.

**Figure 6:**
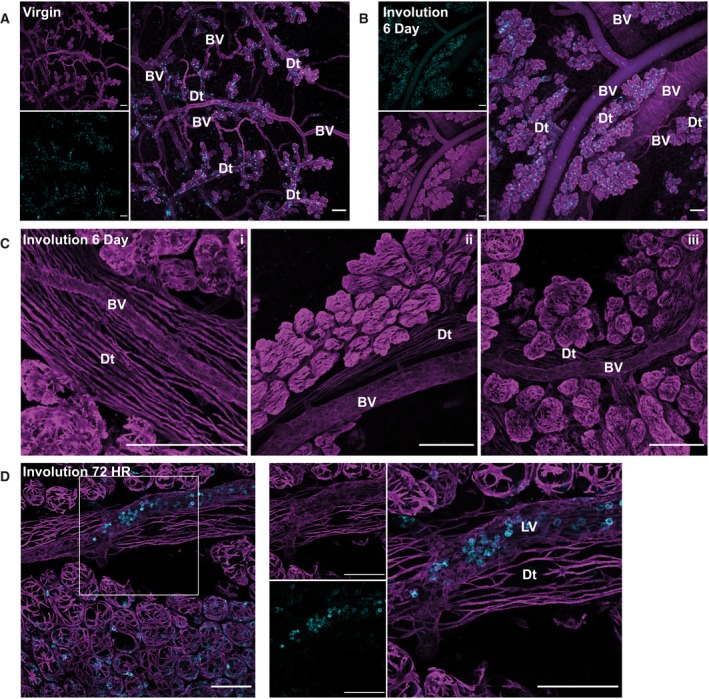
Examples of vascular staining using 3D imaging of the adult mammary gland. Blood and lymphatic vessels were detected using smooth muscle α‐actin (SMA) staining revealing the intricate, and contrasting, pattern of the actin cytoskeleton. Mammary glands from adult BALB/c mice harvested at the indicated involution time points were optically cleared and imaged in 3D using confocal microscopy. All images show 3D maximum intensity projections (MIPs) of the entire optical sequence captured; staining for SMA (magenta), DAPI (grey) and CD45 (cyan). (A, B) Low magnification overviews showing multiple vessel types present in the same field of view in (A) virgin and (B) 6‐day involuted glands; smaller images show individual stains of the merged image in the main panel; (C) i–iii examples of the close association between the vasculature and mammary ducts; (D) a vessel containing many CD45+ cells sitting adjacent to the duct; the boxed region in the left panel is enlarged in the right panel; individual stains are shown to the left of the merged image. Images are representative of at least 4 mice for each time point shown; all scale bars represent 100 μm; Dt, mammary duct; BV, blood vessel; LV, putative lymphatic vessel.

Finally, as we have previously shown, this imaging approach enables the visualisation of tissue vasculature. In virgin glands and during involution, a close juxtaposition of vascular (Fig. 6A–C) and putative lymphatic networks (Fig. 6D) is apparent. This highlights the potential of deep tissue imaging for investigating the interactions between the epithelial tissue and components of the microenvironment.

## Discussion

The interplay between epithelial cells and immune cells in tissues such as lung and gut is well established. In mammary gland, while immune cells have been observed to be localised in close proximity to basal epithelium and in mammary tumours within the tumour epithelium, a comprehensive study using deep imaging has not been previously undertaken. Using our established protocols to address this issue, we made a number of notable, and unexpected, observations. The first of these is the dramatic difference in the morphology of both the myoepithelial and CD45+ cells at different stages of postnatal development. In virgin glands, the basal cells are aligned parallel to each other and in the direction of ductal elongation. Reorientation of this myoepithelial cell layer occurs at branch points and at the ductal tips. In this context, it is worth noting that myoepithelial cells have been shown to suppress the invasion of tumour cells possibly through the production of proteases and antiangiogenic factors 53 and can restrain the escape of tumorigenic luminal cells in organoid cultures 54. Furthermore, while the myoepithelial cell layer is intact in ductal carcinoma *in situ* (DCIS), loss of myoepithelial cells is associated with invasive breast cancer. These findings suggest that myoepithelial cells are dynamically involved in maintaining the architecture of the normal gland, a notion that is supported by our observation of the dynamic change in shape of myoepithelial cells as involution progresses. Our ability to visualise intact ductal structures should facilitate analysis of the role of myoepithelial cell loss in the transition of DCIS to invasive breast cancer.

CD45+ cells are distributed throughout the stroma as expected but also appear attached to the ducts within the epithelial bilayer, where most lie between the basal and luminal epithelial cells. The considerable number of intercalated cells in the ducts of virgin mice raises the question as to the origin of these CD45+ cells. It could be argued that the number of leucocytes, and their pattern of distribution, suggests that the intercalated CD45+ cells arise from a pool of immune cell progenitors that are resident in the mammary gland and proliferate along with the epithelial cells as the duct elongates. Indeed, proliferation of CD11c+ antigen‐presenting cells has been shown to occur in mammary organoids over a period of 15 days in culture 42. However, orchestrated recruitment from the periphery or stroma under the influence of the exquisitely balanced cytokine milieu of the mammary microenvironment is an alternative hypothesis requiring investigation. What is irrefutable is that the rapidly changing epithelial branching morphology during oestrous and pregnancy cycles requires a dynamic response in the immune cell compartment. Leucocytes are essential for postnatal development of the mammary gland as their depletion, by irradiation, results in a failure of normal glandular development 16.

The intercalation of immune cells within the bilayered mammary epithelium raises the question as to their function, which we consider likely multifactorial. One possibility is that immune cells provide immune surveillance as the ducts are open to the environment through the nipple region allowing infiltration of bacteria and other infectious or noxious agents should there be a breach of the innate immune barrier of the teat canal. Another possibility is the production of cytokines that are required for ductal outgrowth.

In lactating mammary glands, we observed that the morphology of both the myoepithelial and CD45+ cells is dramatically different from their counterparts in the ducts of virgin mice. The basal cells no longer align but adopt a stellate shape that forms a complex web overlying the alveolar luminal cells in each bud. While this morphology has been described before 35, we were surprised to observe that the majority of the CD45+ cells have the same shape. While some more rounded cells were apparent, frequently the CD45+ cells closely mirrored the overlying myoepithelial cell. This could suggest that these cells are in physical contact through receptor binding and may communicate directly or indirectly via paracrine signalling. We note that the cytoplasmic processes appear to protrude through the luminal layer where they will be able to sample the alveolar lumen. The expression of MHCII on many of these cells suggests that they are active antigen‐presenting cells. Progesterone has been shown to regulate the Th1/Th2 phenotype of T cells and induces Th2 cytokines during pregnancy 55. Interestingly, although alveologenesis is delayed in pregnancy, lactation occurs normally in mice that are deleted for Stat6 or double‐deleted for IL‐4 and IL‐13, suggesting that Th2 cytokine signalling is not required for lactation 56. In contrast, Th1 signalling and CD4+ T helper cells are required for development during puberty 42.

Involution of the mammary gland is associated with the upregulation of Stat3 signalling and NF‐κB signalling that are undetectable in late lactation, and a dramatic increase in the expression of acute‐phase response genes such as serum amyloids A1 and A2 and orosomucoids, many of which are Stat3 targets 10, 57. Furthermore, previous microarray analyses of involution time points by us and others revealed upregulation of immune mediators during postlactational regression 25, 26. The massive cell death that occurs during the first 4−5 days of involution requires the rapid removal of dead cells, milk fat globules and milk protein, and, following the initial reversible cell death phase, an influx of macrophages occurs around 72‐h involution. Prior to this, the luminal mammary epithelial cells undergo a cell fate switch and become nonprofessisonal phagocytes (or efferocytes) and these cells are responsible for the uptake of milk constituents and dead luminal cells, a process that usually results in their demise 5. It is thus not surprising that the morphology, and possibly the identity, of the CD45+ immune cell population changes as involution progresses and may reflect the changing populations of immune cells that have been described 10, 22.

The few studies that have been carried out on mammary gland blood and lymphatic vasculature have shown that both vessel types are closely apposed to the mammary ducts with the former also penetrating the alveolar buds while the lymphatic network does not 58. Neolymphangiogenesis has been shown to occur during involution of the mammary gland in concert with upregulation of VEGF‐C, VEGF‐D and their receptors 59. A recent study showed that the GPI‐anchored membrane glycoprotein semaphorin 7A (SEMA7A) is expressed in the mammary epithelium during involution and suggested that SEMA7A may play a role in macrophage‐mediated lymphangiogenesis 60. There is, however, some discrepancy in the literature with different investigators reporting maximum lymphatic vessel density at different stages of the pregnancy cycle 58, 61. We therefore propose that tissue clearing be utilised in future studies to properly assess the nature of the relationship between the mammary epithelium and its vasculature, which is only revealed by deep tissue imaging. Our observation that vascular networks run along the mammary ducts (Fig. 6C) is in accord with ultrastructural studies in rat mammary glands 62.

This study has highlighted the level of detail that is missed in conventional histological studies and reveals the necessity for deep 3D imaging in intact tissue to fully appreciate complex interactions between tissue structures, particularly those undergoing dramatic changes in response to hormonal fluctuations. We anticipate that our work will lay the foundation for further studies that address the interplay between mammary epithelium and its environment and how this changes during cycles of development in the adult and during the progression of breast tumorigenesis.

## Materials and methods

### Reagents

The following reagents were purchased from (Sigma‐Aldrich, Company Ltd., Gillingham, UK) : PBS, sodium azide, neutral buffered formalin (NBF), NaCl, urea, *N*,*N*,*N*′,*N*′‐tetrakis(2‐hydroxypropyl)ethylenediamine, sucrose, 2,2′,2″‐nitrilotriethanol and 3′‐diaminobenzidine (DAB). Triton X‐100 was purchased from (VWR International, Lutterworth, UK). Imaging dishes were purchased from Ibidi (81158). Normal goat serum (NGS) was purchased from (Abcam plc, Cambridge, UK) (ab7481). 4′,6‐Diamidino‐2‐phenylindole, dilactate (DAPI) was purchased from (Invitrogen, Scotland, UK) (D1306).

### Animals

BALB/c mice were purchased from Charles River, Harlow, UK, at 6–7 weeks of age. C57BL/6 mice were bred in‐house at the Biological Services Unit, Department of Pathology, University of Cambridge. Unless stated, all mice used were BALB/c. Animals were housed in individually ventilated cages under a 12:12‐hour light/dark cycle with food and water available *ad libitum*. Experiments were performed according to the Animal (Scientific Procedures) Act 1986, and the European Union Directive 86/609, and were approved by the local ethics committee. Group sizes were not predetermined using any statistical methods.

### Involution studies

Females (8 weeks old) were mated in trios using C57BL/6J male studs; after 2 weeks, males were removed, and females were housed individually. Litters were normalised within 3 days of birth to 6–9 pups per dam. After 10 days (range 9–11) of lactation, pups were removed and killed. Dams were cohoused during the involution period and were harvested after 1–11 days. All mice were killed by dislocation of the neck. Excised mammary glands were spread on Tetra Pak card and fixed in 10% NBF overnight at 4 °C. Fixed tissues were stored in PBS containing sodium azide [0.05% (w/v)] for up to 16 weeks. For all virgin time points, mice were 8‐12 weeks old and were not oestrous‐staged. Lactating mice were harvested after 10 days (range 9–11) of lactation.

### Optical tissue clearing and immunohistochemistry

Tissues were optically cleared using the CUBIC protocol as described previously 36. CUBIC reagent 1A contained the following: urea [10% (w/w)], *N*,*N*,*N*′,*N*′‐tetrakis(2‐hydroxypropyl)ethylenediamine [5% (w/w)], Triton X‐100 [10% (w/w)] and NaCl (25 mm) in distilled water; CUBIC reagent 2 contained the following: sucrose [44% (w/w)], urea [22% (w/w)], 2,2′,2″‐nitrilotriethanol [9% (w/w)] and Triton X‐100 [0.1% (w/w)] in distilled water; and blocking buffer contained NGS [10% (v/v)] and Triton X‐100 [0.5% (w/v)] in PBS.

Tissues were cut into small pieces (10 by 10 by 1 mm) and were immersed in CUBIC reagent 1A for 3 days at 37 °C with gentle agitation (solution refreshed daily). Samples were blocked in blocking buffer overnight at 4 °C (with gentle rocking). Primary antibodies were diluted in blocking buffer, and samples were stained for 4 days at 4 °C (gentle rocking). Samples were washed (PBS containing Triton X‐100 (0.1% (w/w))) (3 × 1 h) at room temperature (with gentle agitation) prior to incubation with secondary antibodies for 2 days (4 °C, with gentle rocking). Samples were washed and incubated with DAPI (10 μm) for 2 h (room temperature). Samples were immersed in CUBIC reagent 2 for at least 24 h at 37 °C (with gentle agitation) and were imaged within 1 week. Primary antibodies were omitted from this procedure to ensure detected staining was antibody‐specific.

For two‐dimensional analysis, mammary glands were fixed in 10% NBF and were processed and stained with haematoxylin and eosin (H&E), following standard protocols. For immunohistochemistry, antigen retrieval was performed using a PT‐Link system (Agilent Technologies LDA UK Limited, Life Sciences & Chemical Analysis Group, Stockport, Cheshire, UK) according to the standard procedure.

### Confocal microscopy

Tissues were imaged in Ibidi μ‐dishes using a (Leica SP8 inverted confocal microscope, Milton Keynes, UK) with 10×/0.4 and 20×/0.75 HC PL APO objective lenses. Laser power and gain were manually set for each fluorophore, enabling optimal fluorescence with minimal photobleaching. Images were processed as maximum intensity projections (MIPs) using imagej (version 2.0.0; National Institutes of Health, Bethesda, MD, USA). Image depths are shown from the top of the recorded image (typically 350 μm through the fat pad) 36.

### Antibodies

The following primary antibodies were used for immunostaining of optically cleared tissues: polyclonal rabbit anti‐α‐smooth muscle actin (SMA) (Abcam; ab5694; 1 : 300); and from (BioLegend UK Ltd, London, UK): rat anti‐CD45 clone 30‐F11 (103102; 1 : 300), CD11c clone N418 (117302; 1 : 200) and MHCII I‐A/I‐E clone M5/114.15.2 (107601; 1 : 300). The following macrophage markers were found to be unreliable with the tissue clearing protocols used: rat anti‐F4/80 (clone BM8), rat anti‐CD11b (clone M1/70), rat anti‐CD68 (clone FA‐11) (all from BioLegend) and rat anti‐F4/80 (clone Cl:A3‐1; from AbD Serotec, Kidlington, UK). The following secondary antibodies (all used at 1 : 500) were purchased from Invitrogen: goat anti‐rabbit Alexa Fluor 488 (A11008), goat anti‐rat Alexa Fluor 647 (A21247) and goat anti‐rat Cy3 (A10522); and from (Jackson ImmunoResearch, Ely, UK): goat anti‐Armenian hamster Cy3 (127‐165‐160).

For 2D analysis, SMA expression was detected using a mouse anti‐human SMA primary antibody (clone 1A4, Agilent; M0851) with a peroxidase‐conjugated ImmPRESS anti‐mouse IgG polymer detection kit (Vector Laboratories Ltd; MP‐7402, Peterborough, UK) using standard development with DAB. Mouse IgG1 was used for species‐ and isotype‐matched control (Agilent; X0931).

### Statistics

No statistical analyses were performed.

## Conflict of interest

The authors declare no conflict of interest.

## Author contribution

JRH planned and performed most of the experiments, prepared figures and co‐wrote the manuscript with KH and CJW. OBH and KH contributed to the imaging analysis and the immunohistochemical studies, respectively. All authors analysed data and approved the final manuscript.

## Data Availability

Original confocal image data can be made available by contacting the corresponding author.
